# P-550. Transcriptomic Profiling Reveals Unexpected Similarities Between Bronchiolitis and Alveolar Pneumonia in Young Children Infected with Respiratory Syncytial Virus

**DOI:** 10.1093/ofid/ofaf695.765

**Published:** 2026-01-11

**Authors:** Zhaohui Xu, Sara Mertz, Hannah Kim, Amy Leber, Guy Hazan, Asuncion Mejias, Ron Dagan, Octavio Ramilo

**Affiliations:** St. Jude Children's Research Hospital, Memphis, TN; The Research Institute at Nationwide Children's Hospital, Columbus, Ohio; Research Institute at Nationwide Children's Hospital, Cincinnati, Ohio; Nationwide Childrens Hospital, Columbus, Ohio; Ben Gurion University, Beer Sheva, HaDarom, Israel; St Jude Children's Research Hospital, Memphis, TN; Ben-Gurion University of the Negev, Beer Sheva, HaDarom, Israel; St. Jude Children's Research Hospital, Memphis, TN

## Abstract

**Background:**

Bronchiolitis (BRO) and Alveolar Pneumonia (PNE) are common lower respiratory infections (LRI) leading to hospitalization in children. BRO is generally caused by viruses while PNE is caused by viruses and bacteria. We analyzed transcriptomic profiles in children with LRI to investigate the differences on the immunopathogenesis of BRO vs PNE

**Table 1.**
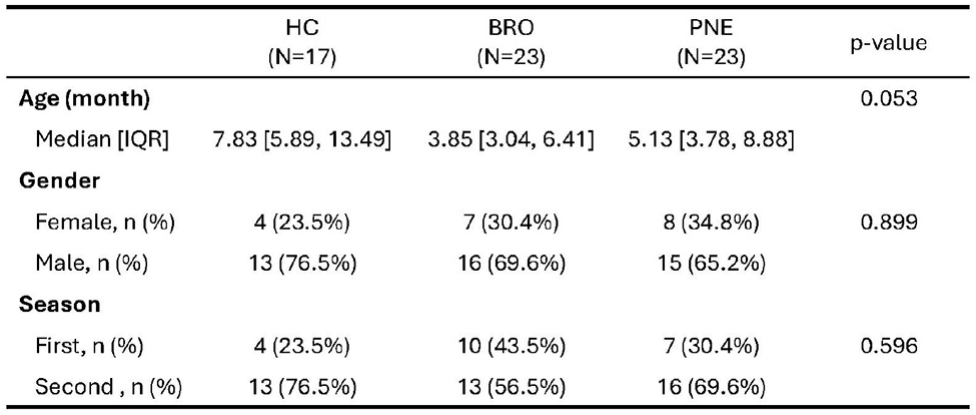
Demographic Characteristics of Study Population

**Figure 1.**
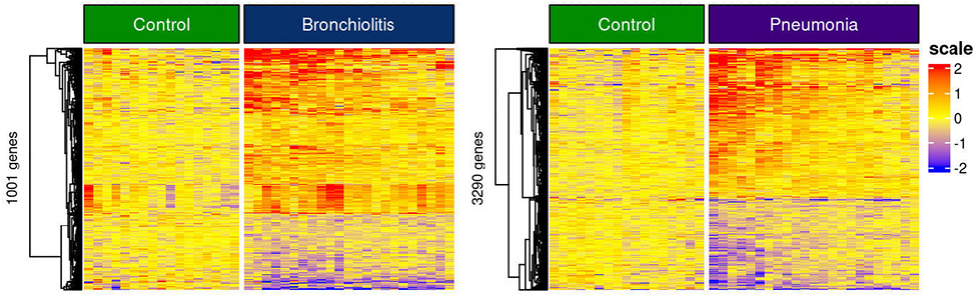
Transcriptomic profiling of LRI samples DEGs were identified by comparing BRO (left) and PNE (right) patient samples vs HC (padj < 0.05 and fold change > 1.25). Heatmaps showed gene expression levels after normalization with median of HC. Red: over-expression, blue: under-expression

**Methods:**

Children < 2yr hospitalized with LRI and healthy controls (HC) were prospectively enrolled during two respiratory seasons (2018-2020) in Beer-Sheva, Israel. Diagnosis of BRO vs PNE was made according to strict predefined criteria. Nasopharyngeal (NP) swabs were analyzed for viruses (Filmarray) and bacteria (qPCR). Blood samples were collected for transcriptome analysis through RNA sequencing and data analysis performed with R

**Figure 2.**
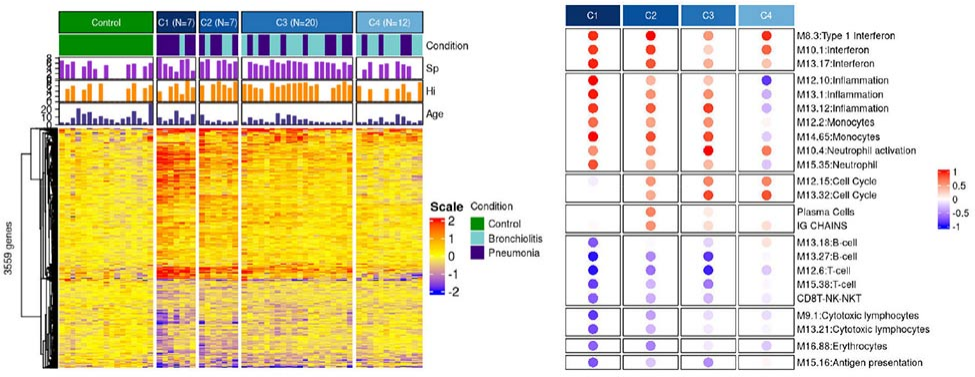
Unsupervised clustering of LRI patient samples

Machine learning approach (K-means clustering) was applied and grouped all LRI samples into 4 clusters (left). The 4 LRI clusters show the individual cases of BRO (light blue) and PNE (dark blue). Bacterial loads (PCR copy number) for Sp (purple) and Hi (orange) and ages (months) are shown on top of the heatmaps. The functional annotation of immune genes of each cluster was illustrated with a modular map, showing the percentage of significant genes in each gene module-set compared with HC (right panel). Red dot: over-expression, blue dot: under-expression

**Figure 3.**
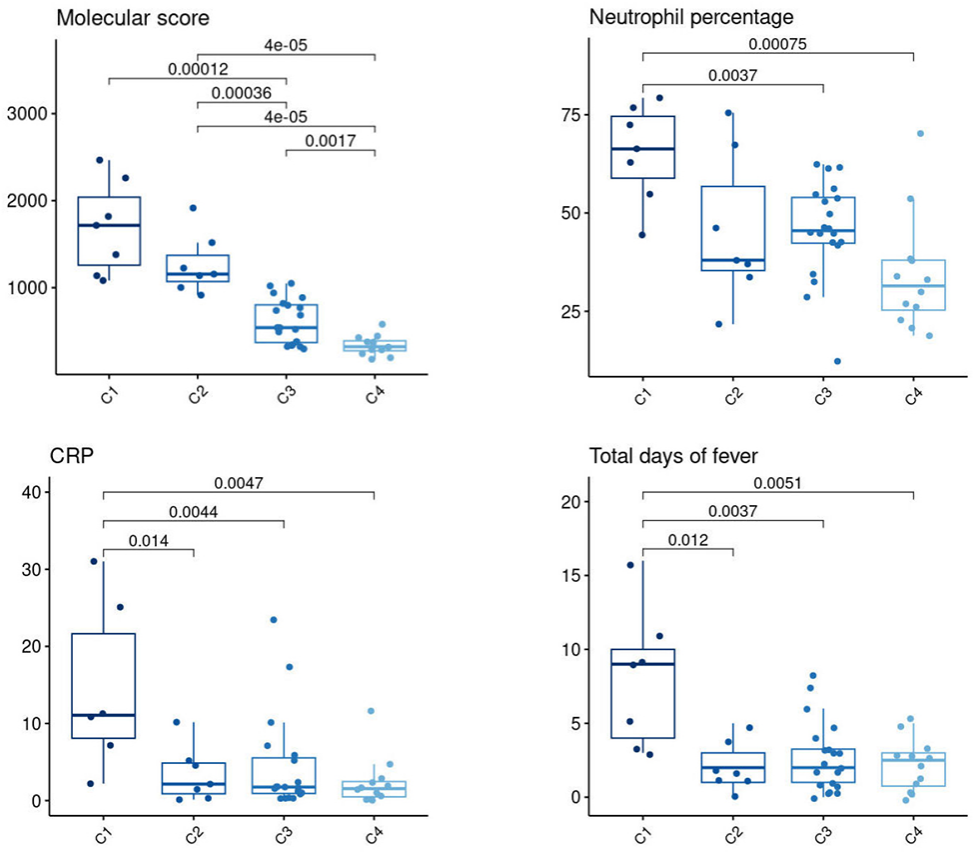
Molecular scores, Neutrophil percentage, CRP values and Total days of fever in all 4 clusters

Transcriptomic scores, along with clinical findings were plotted for the 4 clusters identified by unsupervised analysis. P-values were shown for pairwise comparisons

**Results:**

We enrolled 71 children diagnosed with BRO=35 and PNE=36, and 17 HC. RSV was detected in 23 BRO and 23 PNE patients.

Comparison of RSVBRO and RSVPNE vs HC identified 1,001 and 3,290 differentially expressed genes (DEGs), respectively (Fig 1). Direct comparison between RSVBRO vs RSVPNE identified only 38 DEGs.

Using an unsupervised machine learning approach on a combined gene list from both conditions (n=3,559), we identified four distinct clusters of LRI patients with 7, 7, 20, and 12 patients in each cluster (Fig2). Cases of RSVBRO and RSVPNE were distributed across all four clusters (C), indicating a continuum rather than distinct profiles between the two conditions. There were no significant differences in NP bacterial detection particularly for *S. pneumoniae* (*Sp*) and *H. influenzae* (*Hi*), among all 4 clusters. Functional analysis revealed increased interferon responses in all 4 clusters, but stronger in C1 and C2. C1 exhibited higher inflammation profiles and greater suppression of adaptive immune responses, alongside with higher molecular scores, % of blood neutrophils, CRP levels, and total days of fever (Fig 3)

**Conclusion:**

Transcriptomic analysis comparing children with RSV-associated LRI vs HC identified more DEGs in PNE than in BRO. However, when directly compared, the differences were minimal. Unsupervised clustering demonstrated overlapping gene expression patterns among the LRIs, suggesting a lack of clear distinction in the immunopathogenesis of RSVBRO vs RSVPNE

**Disclosures:**

Amy Leber, PhD, bioMerieux: Grant/Research Support|Biorad: Advisor/Consultant|Qiagen: Advisor/Consultant|Qiagen: Grant/Research Support Guy Hazan, Dr. MD, Sanofi: Grant/Research Support Asuncion Mejias, MD, PhD, MsCS, Enanta: Advisor/Consultant|Merck: Grant/Research Support|Moderna: Advisor/Consultant|Pfizer: Advisor/Consultant|Sanofi-Pasteur: Advisor/Consultant Ron Dagan, Professor MD, GSK: Advisor/Consultant|GSK: Honoraria|Medimmune/AstraZeneca: Grant/Research Support|Medimmune/AstraZeneca: Honoraria|MSD: Advisor/Consultant|MSD: Grant/Research Support|MSD: Honoraria|Pfizer: Advisor/Consultant|Pfizer: Grant/Research Support|Pfizer: Honoraria|Sanofi pasteur: Honoraria Octavio Ramilo, MD, Merck: Advisor/Consultant|Merck: Grant/Research Support|Merck: Honoraria|Moderna: Advisor/Consultant|Pfizer: Advisor/Consultant|Pfizer: Honoraria|Sanofi: Advisor/Consultant

